# Parameter-Efficient Fine-Tuning for Photovoltaic Cell Defect Classification: A Systematic Comparison of LoRA, QLoRA, and Full Fine-Tuning on ConvNeXt-Tiny

**DOI:** 10.3390/s26123659

**Published:** 2026-06-08

**Authors:** Seda Bayat Toksöz, Gültekin Işık, Gökhan Şahin, Erdal Akin

**Affiliations:** 1 Department of Computer Engineering, Iğdır University, Iğdır 76000, Türkiye; seda.bayat@igdir.edu.tr (S.B.T.); gultekin.isik@igdir.edu.tr (G.I.); 2 Municipality of Dronten, De Rede, 1, 8251 ER Dronten, The Netherlands; 3 Copernicus Institute of Sustainable Development, Utrecht University, Princetonlaan 8A, 3584 CB Utrecht, The Netherlands; 4 Department of Computer Science and Media Technology, Malmö University, 205 06 Malmö, Sweden; 5 Sustainable Digitalisation Research Centre, Malmö University, 205 06 Malmö, Sweden; 6 Biofilms Research Center for Biointerfaces (BRCB), Malmö University, 205 06 Malmö, Sweden; 7 Department of Computer Engineering, Bitlis Eren University, Bitlis 13100, Türkiye

**Keywords:** parameter-efficient fine-tuning, LoRA, QLoRA, photovoltaic defect classification, solar cell inspection, ConvNeXt, transfer learning

## Abstract

Automated visual inspection of photovoltaic (PV) cells is an important component of solar-module quality assurance. However, adapting modern pre-trained vision backbones to PV defect classification remains challenging because full fine-tuning requires substantial memory, naturally imbalanced datasets can reduce sensitivity to rare defect classes, and edge-oriented inspection workflows impose computational constraints. Parameter-efficient fine-tuning (PEFT) methods, such as Low-Rank Adaptation (LoRA) and Quantized LoRA (QLoRA), have been widely studied in natural language processing, but their use for PV defect classification remains underexplored. This study presents a controlled benchmark of LoRA and QLoRA against full fine-tuning for PV cell defect classification. Four adaptation strategies—full fine-tuning, LoRA with rank 8, LoRA with rank 16, and 4-bit QLoRA with rank 16—are evaluated using a ConvNeXt-Tiny backbone on a 17,377-image polycrystalline PV cell electroluminescence dataset referred to as POLY, covering five classes: intact, cracked, broken, surface-diffuse, and surface-point. The natural 6.7× class imbalance is preserved without synthetic resampling, and a group-aware StratifiedGroupKFold protocol based on available cell or panel-image identifiers is used to reduce identifiable leakage across folds. All PEFT variants slightly outperform full fine-tuning in macro-F1 while training 26–52× fewer parameters. QLoRA_r16 achieves the highest macro-F1 score of 79.92 ± 0.75%, compared with 78.26 ± 0.94% for full fine-tuning, while training the same number of parameters as LoRA_r16 (1.060 M; 3.67% of the adapted model). QLoRA_r16 also improves F1 on the intact (+4.75 points) and surface-diffuse (+2.62 points) classes relative to full fine-tuning. This class-wise pattern suggests that quantized low-rank adaptation may influence minority and visually ambiguous categories; however, the present experiments do not isolate the independent effect of NF4 quantization from adapter rank, batch size, or optimization dynamics. Under the training configuration used, QLoRA_r16 records the lowest observed peak training GPU memory, approximately 30% below full fine-tuning (1727 MB versus 2478 MB). Because QLoRA_r16 was trained with batch size 16 whereas the other methods used batch size 32, this reduction should be interpreted as an end-to-end configuration effect rather than as the isolated effect of 4-bit quantization. Overall, the results indicate that PEFT is a promising and resource-efficient alternative to full fine-tuning for PV defect classification, although batch-matched memory experiments, direct embedded-device profiling, and cross-dataset validation remain necessary before making deployment-level claims.

## 1. Introduction

The global deployment of photovoltaic (PV) technology has accelerated rapidly over the last decade, with PV now recognized as a terawatt-scale energy technology and with cumulative installed capacity exceeding 2.2 TW globally by the end of 2024 [1,2]. As the installed capacity of PV systems continues to increase, maintaining the reliability and operational efficiency of solar modules has become increasingly important. Defects introduced during wafer processing, cell manufacturing, module assembly, transportation, or long-term field operation can adversely affect energy production and system lifetime. Previous studies have shown that micro-cracks and broken cells can significantly reduce module performance and accelerate degradation processes [3]. In addition, diffuse surface defects and point-like surface anomalies may evolve into more severe failures and substantial power losses when they remain undetected [4]. Consequently, accurate and automated defect inspection has become a critical component of modern photovoltaic manufacturing and maintenance processes. Among the available inspection techniques, electroluminescence (EL) imaging has proven particularly effective for identifying structural defects that are difficult to observe using conventional visual inspection methods [5]. Its capability to reveal electrically inactive regions and subsurface imperfections has made EL imaging a standard tool in photovoltaic quality assessment [6]. The ability of EL imaging to expose hidden defects has stimulated considerable interest in the development of deep learning-based computer vision systems for automated PV defect classification. ResNet-based transfer learning approaches have demonstrated strong performance in photovoltaic defect recognition tasks [7]. Similar success has been reported using VGG and DenseNet architectures for EL image analysis [8]. Attention-enhanced convolutional networks and SENet-based approaches have further improved defect discrimination capabilities [9]. Inception-based models have also shown competitive classification performance in photovoltaic inspection applications [10]. More recently, Vision Transformer architectures have emerged as powerful alternatives capable of capturing long-range spatial relationships in EL images [11]. Most of these approaches rely on full fine-tuning, where all parameters of a pre-trained model are updated using task-specific training data. Although full fine-tuning often achieves strong predictive performance, several practical challenges arise when deploying such models in industrial environments. First, updating all model parameters requires substantial computational resources and memory, which can limit scalability across multiple production lines. Second, photovoltaic defect datasets are typically characterized by significant class imbalance, where rare but operationally important defect classes are represented by relatively few samples. Under these conditions, full fine-tuning may over-adapt to dominant classes while providing limited improvements for minority classes. Third, the growing use of embedded artificial intelligence hardware for real-time inspection introduces strict memory and computational constraints that are not always compatible with conventional fine-tuning strategies. To address similar limitations in other machine learning domains, parameter-efficient fine-tuning (PEFT) methods have recently attracted considerable attention. Rather than updating an entire pre-trained network, PEFT methods optimize only a small number of additional trainable parameters while keeping most of the backbone frozen. Low-Rank Adaptation (LoRA) introduces trainable low-rank matrices to represent task-specific weight updates, thereby reducing the number of parameters that must be optimized [12]. Quantized LoRA (QLoRA) further extends this concept by combining low-rank adaptation with low-bit quantization, significantly lowering memory requirements while maintaining competitive performance [13]. Similar ideas have been adopted in computer vision through Visual Prompt Tuning [14], AdaptFormer [15], and LoRA-based adaptation strategies for Vision Transformers [16]. Despite the rapid adoption of PEFT methods in natural language processing and general computer vision tasks, their application to photovoltaic defect classification remains largely unexplored. Earlier photovoltaic defect studies mainly focused on conventional transfer learning frameworks based on CNN architectures [7]. Subsequent investigations explored deeper and more sophisticated architectures to improve classification accuracy [8]. Attention-based networks and feature-enhancement mechanisms have also been proposed for defect identification [9,17]. Transformer-based approaches have recently attracted increasing attention because of their ability to model complex spatial dependencies within EL images [18]. Other studies have investigated lightweight architectures for industrial deployment scenarios [19]. Nevertheless, these approaches generally depend on full fine-tuning of the backbone network [20]. Furthermore, recent reviews of photovoltaic inspection techniques provide only limited discussion of parameter-efficient adaptation strategies [5]. As a result, it remains unclear whether low-rank adaptation can provide sufficient representational capacity for photovoltaic defect recognition while reducing computational complexity. The influence of quantization on minority-class performance has also not been systematically investigated, despite class imbalance being recognized as a major challenge in photovoltaic defect classification [11,20]. Motivated by these observations, this study investigates the effectiveness of parameter-efficient fine-tuning strategies for photovoltaic defect classification using electroluminescence images. We perform a systematic comparison between conventional full fine-tuning and PEFT-based approaches, including LoRA and QLoRA, under identical experimental conditions. The objective is to evaluate their impact on classification performance, minority-class recognition, memory efficiency, and deployment suitability, while providing new insights into the applicability of parameter-efficient learning techniques for industrial photovoltaic inspection systems. The present study is designed as a controlled benchmark rather than as a new architecture proposal. Its contribution is threefold. First, it evaluates full fine-tuning, LoRA_r8, LoRA_r16, and QLoRA_r16 under a shared ConvNeXt-Tiny backbone, identical preprocessing, a class-weighted loss, and a five-fold evaluation protocol. Second, it analyzes not only average macro-F1 but also class-wise F1, recall behavior, fold stability, train–validation gap, trainable parameter count, training memory, and inference latency. Third, it explicitly reports the validation boundaries of the benchmark, including the proprietary nature of the POLY dataset, the near one-to-one group identifier structure, the non-batch-matched QLoRA memory comparison, and the workstation-based nature of the efficiency profiling. These design choices allow the results to be interpreted as controlled within-dataset benchmark evidence for PEFT in PV defect classification, while avoiding unsupported claims about fully module-independent generalization, embedded-device readiness, or architecture-independent superiority.

## 2. Related Work

### 2.1. Deep Learning for Photovoltaic Defect Classification

The increasing adoption of electroluminescence (EL) imaging in photovoltaic quality control has encouraged the development of deep learning techniques for automated defect recognition. One of the most influential contributions in this field was presented by Deitsch et al. [7], who introduced the ELPV benchmark dataset and demonstrated the feasibility of applying deep learning methods to classify photovoltaic cell defects. Their work established a common evaluation framework that has been widely adopted by subsequent studies. Following this direction, Akram et al. [8] explored transfer learning with convolutional neural networks and demonstrated that high classification accuracy could be achieved even with relatively lightweight architectures. Their findings highlighted the practical value of pre-trained models when only limited photovoltaic training data are available. As research progressed, investigators began to explore more sophisticated architectures. For example, Wang et al. [9] integrated coordinate-attention mechanisms into a hybrid framework combining ResNet-152 and Xception networks. Their results suggested that attention-based feature extraction can improve robustness when dealing with class-imbalanced defect datasets. The effectiveness of ensemble learning has also been investigated. Tella et al. [10] evaluated a diverse collection of deep learning architectures, including CNNs and Vision Transformers, and combined their outputs through voting and bagging strategies. Although the ensemble approach improved overall stability, the reported results indicated that photovoltaic defect classification remains a challenging task, particularly when multiple defect categories are considered simultaneously. Similarly, Ebied et al. [11] addressed the class imbalance problem through generative data augmentation and optimized learning rate scheduling, demonstrating that training strategies can be as important as architectural choices. Object detection approaches have attracted increasing attention in recent years. Cao et al. [17] proposed an enhanced YOLOv8-based framework specifically designed for photovoltaic defect localization, incorporating depthwise separable convolutions and feature fusion mechanisms to improve detection efficiency. Transformer-based approaches have also emerged as promising alternatives. The PD-DETR framework introduced by Wang et al. [18] combined transformer-based set prediction with enhanced feature extraction modules, while PV-YOLOv12n [19] incorporated attention-enhanced structures to improve crack detection performance in photovoltaic modules. Al-Waisy et al. [21] further explored deep feature representations for identifying defective solar cells in EL images. In another comparative study, Al-Otum [22] systematically evaluated multiple CNN architectures and highlighted the importance of selecting an appropriate network design according to defect characteristics and image quality. Although these studies collectively demonstrate substantial progress in photovoltaic defect recognition, most of them share a common characteristic: they rely on either full fine-tuning of large pre-trained networks or complete training of custom architectures. Consequently, model adaptation often requires considerable computational resources. Furthermore, many published studies evaluate performance using random image-level splits or private datasets, making it difficult to assess the influence of data leakage arising from highly correlated samples originating from the same photovoltaic module. These limitations motivate the investigation of alternative adaptation strategies that can maintain competitive performance while reducing computational overhead.

### 2.2. Parameter-Efficient Fine Tuning

Parameter-efficient fine-tuning (PEFT) has recently emerged as an attractive alternative to conventional full-network optimization. The central idea behind PEFT is that large pre-trained models already contain rich and transferable representations, making it unnecessary to update every parameter for a new downstream task. Instead, only a small subset of additional parameters is trained while the original backbone remains largely frozen. Several PEFT strategies have been proposed in the literature. Adapter-based approaches introduce lightweight bottleneck layers into pre-trained architectures, allowing task-specific information to be learned with minimal parameter updates [23]. Other methods, including prompt tuning [24] and prefix tuning [25], adapt model behavior by learning trainable input representations rather than modifying the backbone weights directly. Among the available techniques, Low-Rank Adaptation (LoRA) has attracted particular interest due to its simplicity and effectiveness [12]. LoRA represents task-specific weight modifications through low-rank matrix decompositions, dramatically reducing the number of trainable parameters. An important practical advantage is that the learned updates can be merged with the original model during inference, eliminating additional computational cost. Building upon this concept, QLoRA further reduces memory requirements by combining low-rank adaptation with 4-bit NormalFloat quantization and memory-efficient optimization strategies [13]. Originally developed for large language models, QLoRA demonstrated that high-quality adaptation can be achieved with substantially lower hardware requirements.

### 2.3. Peft in Computer Vision and Pervasive Sensing

Although PEFT techniques were initially developed within natural language processing, their application has rapidly expanded into computer vision. Visual Prompt Tuning introduced the concept of adapting Vision Transformers through a small set of learnable visual prompts rather than modifying the backbone parameters [14]. Similarly, AdaptFormer proposed lightweight adapter branches that enable efficient adaptation across multiple visual recognition tasks while preserving most of the original network weights [15]. Subsequent studies demonstrated that LoRA-based adaptation can be successfully applied to image classification, segmentation, and generative vision tasks [16,26]. These investigations consistently reported that only a small fraction of trainable parameters is often sufficient to achieve performance comparable to conventional full fine-tuning. Such findings suggest that much of the representational power required for downstream visual tasks is already encoded within large pre-trained models. Beyond traditional computer vision, PEFT has also shown promise in pervasive sensing applications. Seregina et al. [27] reported that LoRA and QLoRA achieved competitive performance in human activity recognition tasks while substantially reducing training complexity. Their results indicate that parameter-efficient adaptation may be particularly beneficial in domains where computational resources are limited and datasets are relatively specialized. Despite these encouraging developments, evidence regarding the effectiveness of PEFT in renewable-energy applications remains scarce. In particular, photovoltaic defect classification presents unique challenges arising from subtle visual differences between defect categories, strong class imbalance, and the need for deployment on resource-constrained industrial hardware. To the best of our knowledge, a systematic comparison of LoRA- and QLoRA-based adaptation strategies for photovoltaic defect classification has not yet been reported. The present study seeks to address this gap by providing a comprehensive evaluation of parameter-efficient fine-tuning methods within a realistic photovoltaic inspection setting.

Recent sensing and materials-oriented studies also show that photovoltaic inspection and energy-harvesting systems are influenced not only by classification algorithms but also by device-level optical, coating, and monitoring technologies. For example, Oliva et al. [28] investigated zeolite-based anti-reflective coatings for implantable bioelectronic devices and showed that surface-engineering strategies can reduce optical reflection and improve photovoltaic conversion-related behavior. Although this line of work is not directly concerned with EL image classification, it highlights the broader sensing and device-level context in which AI-based inspection systems are deployed. In this respect, the present study complements materials- and device-oriented advances by focusing on the training efficiency of vision models used for automated PV defect classification.

## 3. Materials and Methods

This section describes the dataset, model architecture, training strategy, and evaluation protocol used in this study. The overall goal is to establish a controlled and reproducible experimental framework for comparing full fine-tuning with parameter-efficient fine-tuning (PEFT) approaches under identical data and optimization conditions. Particular attention is given to maintaining realistic data distributions, preventing data leakage, and ensuring fair comparisons across all methods.

### 3.1. Dataset

The experiments are conducted on the POLY dataset, which contains 17,377 electroluminescence (EL) images of polycrystalline silicon photovoltaic (PV) cells acquired from an institutional inspection system. Each sample corresponds to an individual solar cell cropped from full module-level EL acquisitions. The dataset is labeled into five defect categories: intact, cracked, broken, surface-diffuse, and surface-point, based on expert-defined industrial inspection criteria. In this labeling scheme, “intact” refers to defect-free cells, “cracked” indicates fine linear micro-fractures, “broken” represents severe structural discontinuities, “surface-diffuse” corresponds to spatially spread low-intensity regions, and “surface-point” denotes localized spot-like anomalies. The dataset exhibits a pronounced class imbalance, with class proportions ranging from 6.89% to 46.08%, corresponding to an imbalance ratio of approximately 6.7×. Instead of applying resampling or synthetic augmentation at the dataset level, the original distribution is preserved to better reflect real-world manufacturing conditions. To compensate for this imbalance during training, inverse frequency class weighting is applied in the loss function. Table 1 presents the dataset composition and computed class weights used during training.

**Figure 1:**
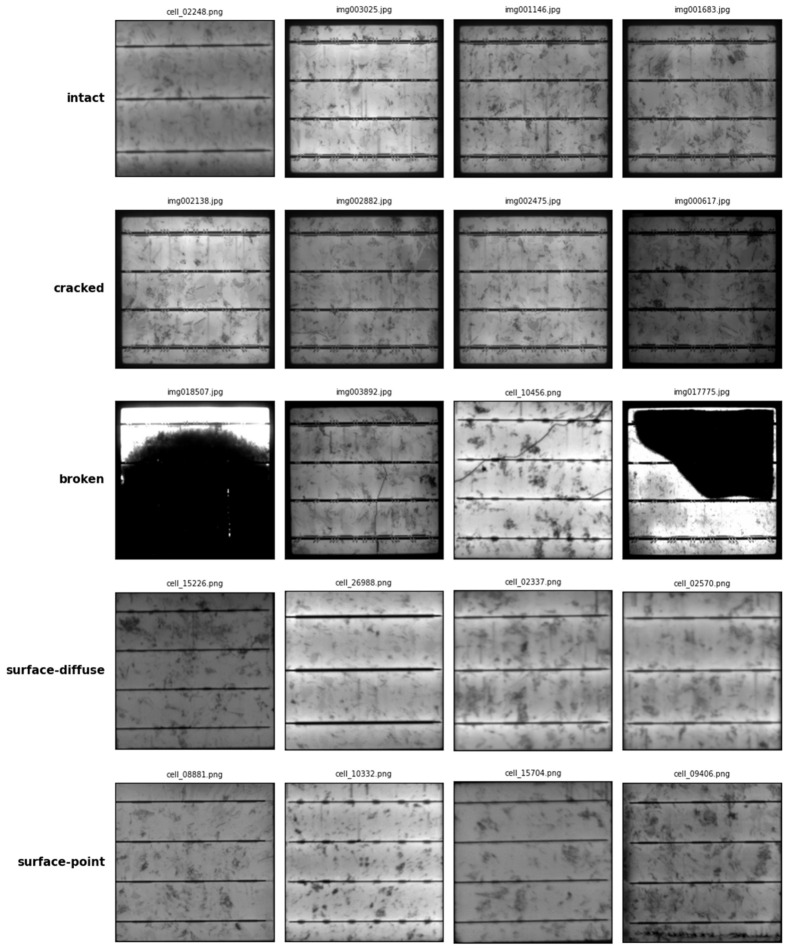
Example EL images from the POLY dataset across five defect categories.

Prior to model input, all images are resized to 240 × 240 pixels, followed by a random crop to 224 × 224 during training. For evaluation, a deterministic resize to 224 × 224 is applied. Data augmentation includes moderate color perturbations (brightness and contrast ±0.25, saturation ±0.15), small spatial shifts of up to 5% of image dimensions, and random erasing with a probability of 0.15. All inputs are normalized using ImageNet-1K (ILSVRC2012) statistics to maintain compatibility with the pre-trained backbone. To avoid leakage between visually similar samples originating from the same physical module, group-based splitting is enforced during cross-validation. Figure 1 illustrates representative samples from each defect class in the dataset.

This figure provides qualitative insight into intra-class variation and inter-class visual differences. Intact cells show uniform emission patterns, while cracked and broken samples exhibit progressively stronger structural discontinuities. Surface-diffuse and surface-point classes reflect distributed and localized intensity anomalies, respectively, which are often challenging to distinguish without domain expertise. The POLY images were obtained from an institutional EL inspection workflow and provided as cropped cell-level grayscale images extracted from larger module-level captures. The available metadata include class labels and group identifiers used for fold construction, but the complete acquisition metadata, including the exact camera model, excitation current, exposure settings, illumination configuration, and original full-module acquisition protocol, cannot be redistributed because of institutional and industrial data-sharing restrictions. To avoid implying a level of reproducibility that cannot be guaranteed from the available metadata, we report the accessible preprocessing, cropping, labeling, class distribution, and fold-construction details explicitly, and we treat the absence of full acquisition metadata as a limitation of the benchmark.

### 3.2. Backbone: Convnext-Tiny

The backbone network used in this study is ConvNeXt-Tiny (ConvNeXt-T) [29], initialized with ImageNet-1K pretrained weights. This architecture contains approximately 27.82 million parameters and follows a hierarchical design composed of four stages of convolutional blocks with increasing channel dimensions. ConvNeXt-T achieves performance comparable to transformer-based architectures such as Swin Transformer [30], while maintaining lower computational complexity. In the context of photovoltaic defect recognition, ConvNeXt-T is particularly suitable due to its ability to capture fine-grained texture variations in electroluminescence images, including subtle intensity gradients, grid-line disruptions, and localized dark regions associated with defects. The model is computationally efficient enough for moderate batch-size training while still providing sufficient representational capacity for fine-grained classification tasks. The original classification head (1000 classes) is replaced with a task-specific fully connected layer producing five output classes, initialized using Kaiming normalization.

### 3.3. Fine-Tuning Methods

#### 3.3.1. Full Fine-Tuning

In the full fine-tuning baseline (full_ft), all parameters of ConvNeXt-T and the classification head are updated during training. This approach results in approximately 27.82 million trainable parameters and serves as the reference method in terms of both performance and computational cost.

#### 3.3.2. LoRa

Low-Rank Adaptation (LoRA) [12] introduces a parameter-efficient mechanism by representing task-specific weight updates through a low-rank decomposition. For a frozen pre-trained weight matrix W0 with dimensions d_out × d_in, LoRA defines the adapted weight as:

W = W0 + ΔW,   ΔW = (α/r) B A(1)

where A has dimensions r × d_in, B has dimensions d_out × r, r is the adapter rank, and α is the LoRA scaling factor. The original weight matrix W0 remains frozen, and only the low-rank matrices A and B are optimized during fine-tuning. This design reduces the number of trainable parameters while preserving the representational capacity of the pre-trained backbone. Within ConvNeXt-Tiny, LoRA is applied to the two pointwise projection layers, denoted as mlp.fc1 and mlp.fc2, inside each ConvNeXt block. These layers are selected because they perform channel mixing and account for a substantial fraction of the adaptation capacity of the architecture. The depthwise convolution, normalization layers, and remaining backbone parameters are kept frozen, while the task-specific classification head is trained. Two LoRA configurations are evaluated: LoRA_r8 with rank r = 8 and α = 16, resulting in 0.530 M trainable parameters, and LoRA_r16 with rank r = 16 and α = 32, resulting in 1.060 M trainable parameters. These correspond to 1.87% and 3.67% of the adapted model parameters, respectively. When referenced against the original full fine-tuning backbone alone, the corresponding shares are approximately 1.90% and 3.81%.

#### 3.3.3. QLoRa

**Figure 2:**
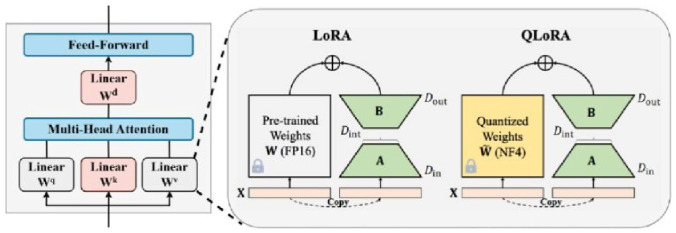
Comparison of LoRA and QLoRA adaptation mechanisms.

QLoRA [13] extends LoRA by introducing low-bit quantization to the frozen backbone. In this work, the pretrained ConvNeXt-T weights are quantized to 4-bit NormalFloat (NF4) precision using bitsandbytes [31], while LoRA adapters remain in bfloat16 format. This design changes the memory profile of the evaluated training configuration without altering the number of trainable parameters; however, the present experiments do not isolate the effect of quantization from the smaller batch size used by QLoRA. For fair comparison, QLoRA is configured with r = 16 and α = 32, matching the LoRA_r16 setup in terms of adapter capacity (1.060 M parameters). The key distinction is therefore not parameter count but numerical precision of the frozen backbone. LoRA maintains full precision (FP16/bfloat16) weights, whereas QLoRA applies NF4 quantization with double quantization of scaling constants. Figure 2 illustrates the conceptual difference between LoRA and QLoRA.

**Figure 3:**
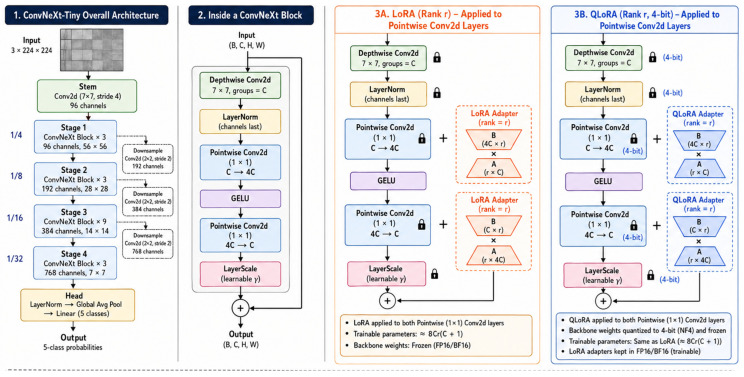
ConvNeXt-Tiny architecture with LoRA and QLoRA injection points.

This diagram shows that both methods preserve the original weight matrix W as frozen and introduce a low-rank trainable branch composed of matrices A and B. The outputs of the frozen and trainable paths are summed. The key difference lies in precision: LoRA retains full-precision weights, while QLoRA applies 4-bit NF4 quantization to the frozen parameters. Figure 3 presents the integration of LoRA/QLoRA into ConvNeXt-T architecture.

The figure illustrates the full network pipeline including stem, four hierarchical stages, and classification head. It further highlights the insertion of low-rank adapters into the two pointwise projection layers of each block. In QLoRA, the same insertion points are used, but the frozen backbone operates in quantized 4-bit precision. This design enables consistent structural comparison between methods while isolating the effect of parameter efficiency and quantization.

### 3.4. Cross-Validation Protocol: Group-Aware Stratifiedgroupkfold

To reduce identifiable leakage between visually related samples, we use a five-fold StratifiedGroupKFold protocol based on the available cell or panel-image group identifiers. The dataset contains 17,377 images and 17,324 unique group identifiers, indicating that most images have unique group IDs and that only a small subset of samples share identifiers with other images. For samples with shared group IDs, the splitting procedure ensures that all images from the same group are assigned entirely to the same fold. Stratification is simultaneously preserved over the five class labels so that each fold approximates the global class distribution. Because the grouping metadata are close to one to one and do not provide complete module level lineage for all samples, this protocol should be interpreted as group-aware and leakage reduced rather than as a fully module independent validation design.

As shown in Table 2, within each fold, the dataset is further divided into training (≈85%) and validation (≈15%) subsets using group-aware splitting. Training is performed for a maximum of 20 epochs with early stopping based on validation macro-F1 (patience = 6, minimum improvement = 0.001). Optimization is performed using AdamW [32] with a weight decay of 0.05, cosine learning rate scheduling, and a short warm-up phase. Class imbalance is handled using weighted cross-entropy, and gradient clipping is applied for training stability.

### 3.5. Evaluation Metrics

Performance is evaluated using multiple complementary metrics: overall accuracy, macro-averaged F1-score, weighted F1-score, and class-wise precision and recall. Results are reported as mean ± standard deviation across five cross-validation folds. In addition to predictive performance, computational efficiency is assessed using three metrics: trainable parameter count, peak GPU memory consumption during training, and inference latency, measured over repeated forward passes. To further analyze generalization behavior, the difference between training and validation macro-F1 scores is also reported as an indicator of overfitting tendency. All experiments were implemented in PyTorch 2.3.1 [33], using timm 1.0.7 [34] for the ConvNeXt-Tiny backbone, scikit-learn 1.5.1 [35] for stratified group-aware fold construction and metric computation, and bitsandbytes 0.43.3 [31] for 4-bit NF4 quantization in the QLoRA configuration.

## 4. Results

This section presents the experimental findings obtained from the comparative evaluation of full fine-tuning and parameter-efficient fine-tuning (PEFT) methods on the ConvNeXt-Tiny backbone. The analysis is structured into four parts: overall performance comparison, class-wise behavior, computational efficiency, and generalization stability across folds. All reported results correspond to 5-fold cross-validation averages with standard deviation values to reflect variability across splits.

### 4.1. Main Comparison

**Figure 4:**
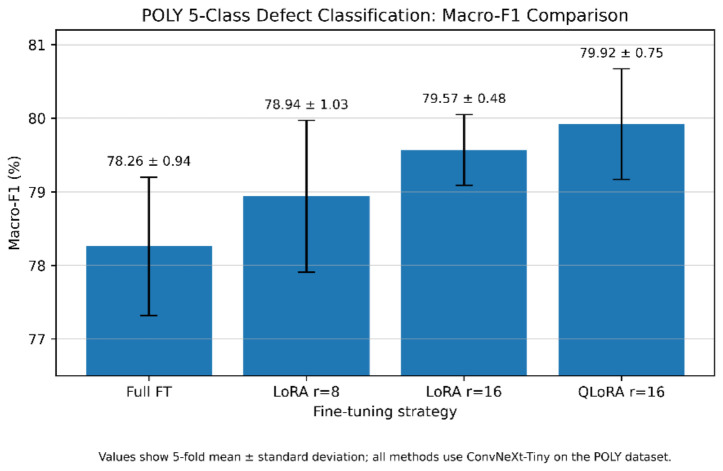
Macro-F1 comparison across the four fine-tuning strategies on ConvNeXt-Tiny using the POLY dataset. Bars indicate the five-fold mean macro-F1, and error bars indicate the standard deviation across folds. All three PEFT variants outperform full fine-tuning under the same dataset and evaluation protocol. QLoRA with rank 16 obtains the highest mean macro-F1 score (79.92 ± 0.75%), although the absolute improvement over full fine-tuning is modest (+1.66 percentage points) and should be interpreted as a controlled within-dataset benchmark result rather than as evidence of deployment-level superiority. The figure shows that PEFT-based approaches obtain slightly higher mean macro-F1 values than full fine-tuning under the evaluated POLY benchmark. QLoRA_r16 achieves the highest average performance, but the absolute difference is modest and should be interpreted within the present single-backbone, within-dataset setting.

The primary quantitative comparison between full fine-tuning, LoRA variants, and QLoRA is summarized in Table 3, while Figure 4 provides a visual comparison of macro-F1 performance across folds. Table 3 reports overall performance, efficiency, and generalization indicators for each method.

Table 3 summarizes the overall classification and efficiency results across the four adaptation strategies. All PEFT configurations improve macro-F1 relative to full fine-tuning, with gains of +0.68 points for LoRA_r8, +1.31 points for LoRA_r16, and +1.66 points for QLoRA_r16. These gains are consistent but modest, which is expected because all methods use the same strong ConvNeXt-Tiny backbone and are trained under a matched data pipeline. Therefore, the main contribution is not a large absolute performance leap, but the observation that parameter-efficient adaptation can match or slightly exceed full fine-tuning while substantially reducing the number of trainable parameters. Increasing the LoRA rank from 8 to 16 improves both mean macro-F1 and fold stability, as reflected by the lower standard deviation of LoRA_r16. QLoRA_r16 achieves the highest mean macro-F1 and is the only configuration with a non-negative train–validation gap. This observation is compatible with a possible regularization-like empirical pattern, but it should not be interpreted as mechanistic evidence that NF4 quantization itself improves generalization. Because the present benchmark does not include ablations that independently vary quantization, adapter rank, batch size, and optimizer dynamics, the result should be interpreted as an empirical pattern observed under the evaluated configuration. Moreover, because the QLoRA run uses a smaller batch size than the other methods, its memory advantage should be interpreted as an end-to-end configuration effect rather than as the isolated effect of NF4 quantization alone.

### 4.2. Per-Class Analysis

**Figure 5:**
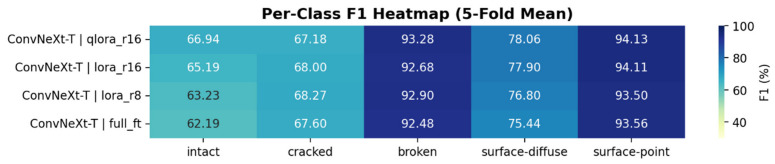
Heatmap of per-class F1 scores (5-fold mean).

To better understand class-specific behavior under imbalance conditions, per-class F1 scores are reported in Table 4, while Figure 5 visualizes these results in heatmap form. Table 4 provides class-wise F1 performance across all methods.

Figure 5 shows the per-class F1 distribution across methods.

The visualization highlights that QLoRA_r16 achieves the strongest performance in most classes, particularly in intact and surface-diffuse categories, while LoRA_r8 shows a slight advantage in the most underrepresented cracked class. The improvements observed for intact and surface-diffuse defects indicate that QLoRA_r16 may alter the class-wise behavior of the model under imbalance. However, these results should not be attributed solely to NF4 quantization, because quantization, low-rank adaptation, batch-size differences, and optimization dynamics are not independently isolated in the present benchmark. In contrast, performance on the cracked class shows a mild reduction under QLoRA compared to LoRA_r8, indicating that no single adaptation configuration dominates across all defect categories.

Table 5 reports the per-class recall values obtained from 5-fold cross-validation for all examined fine-tuning strategies. The purpose of this breakdown is to move beyond single-score evaluation and inspect how each method behaves under different defect characteristics, which is particularly important in a long-tailed industrial dataset such as PV electroluminescence images. Across all models, a clear structural pattern can be observed in the difficulty of classes. The “surface-point” and “broken” categories consistently yield higher recall values, indicating that these defect types contain more discriminative visual cues that are easier for convolutional representations to capture. In contrast, the “intact” and “cracked” classes remain comparatively challenging, with noticeably lower recall and higher variability across folds, suggesting weaker visual separability and higher intra-class diversity. From a method perspective, LoRA-based adaptation, especially at higher rank (r = 16), slightly improves recall in several categories, indicating that restricting updates to a low-rank subspace does not prevent class discrimination. QLoRA_r16 further improves recall in some categories, particularly “intact”, “broken”, and “surface-diffuse”; however, this pattern should be interpreted as configuration-level evidence rather than as proof that NF4 quantization independently improves generalization. The “cracked” class remains an important exception, where LoRA_r8 achieves the highest recall, suggesting that lower-rank adaptation may sometimes better preserve sensitivity to very subtle and data-scarce defect patterns.

**Figure 6:**
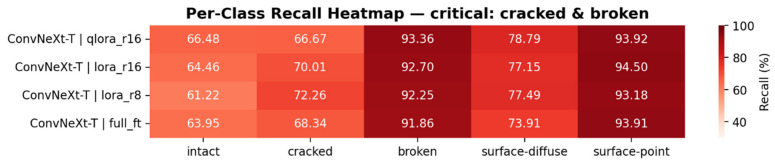
Per-class recall heatmap across the evaluated fine-tuning strategies.

Figure 6 visualizes the same per-class recall results presented in Table 5 using a heatmap representation, which makes cross-method and cross-class comparisons more intuitive. Instead of focusing on numerical differences alone, the figure emphasizes relative intensity patterns across classes and methods. A stable structure emerges across all models: “broken” and “surface-point” classes form consistently high-response regions, indicating that these categories are reliably captured regardless of the fine-tuning strategy. In contrast, “intact” and “cracked” classes remain visually weaker across the heatmap, reflecting their inherent ambiguity and lower feature distinctiveness. QLoRA_r16 exhibits a relatively balanced heatmap profile across several classes, whereas LoRA variants show relatively stronger performance in the “cracked” class. This trade-off highlights that no single configuration dominates across all defect types, and that the observed class-wise behavior should be interpreted as an empirical outcome of the complete training configuration rather than as a mechanism isolated to quantization.

### 4.3. Efficiency Trade-Off

**Figure 7:**
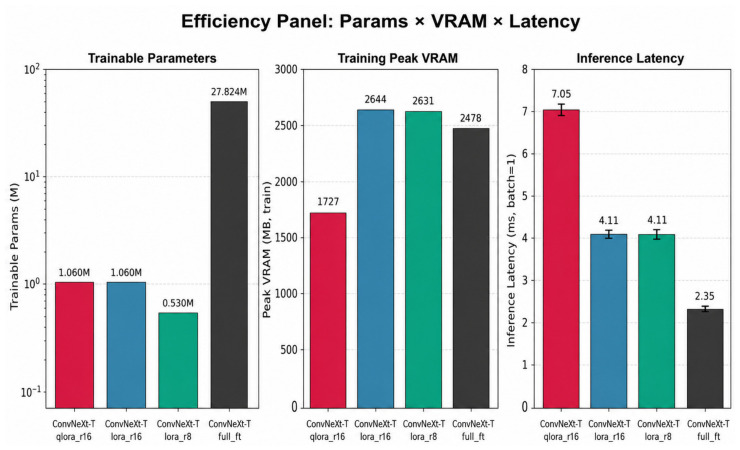
Efficiency comparison of full fine-tuning, LoRA, and QLoRA on ConvNeXt-Tiny. The left panel shows the number of trainable parameters on a logarithmic scale, the middle panel reports peak training GPU memory, and the right panel shows batch-1 inference latency. QLoRA_r16 achieves the lowest observed training memory under the evaluated configuration, but this result is not batch-matched because QLoRA_r16 uses batch size 16 while the other methods use batch size 32. Latency and memory values were measured on an NVIDIA RTX PRO 6000 workstation and should therefore be interpreted as workstation-based profiling results rather than direct embedded-device deployment measurements.

This section examines the computational and deployment-related implications of the four fine-tuning strategies by jointly evaluating model complexity, memory consumption during training, and inference-time latency. The comparison is summarized in Figure 7, which jointly visualizes parameter scale, GPU memory usage, and runtime performance across methods.

Figure 7 presents a three-part efficiency breakdown of the evaluated adaptation strategies. The left panel reports the number of trainable parameters on a logarithmic scale, the middle panel shows peak GPU memory consumption during training, and the right panel reports batch-1 inference latency. This visualization separates the parameter-efficiency profile of full fine-tuning from the PEFT-based configurations, while also showing the different resource trade-offs introduced by LoRA and QLoRA. From a trainable-parameter perspective, full fine-tuning updates the entire ConvNeXt-Tiny backbone, corresponding to approximately 27.8 million trainable parameters. In contrast, LoRA_r8, LoRA_r16, and QLoRA_r16 update only the adapter and classification-head parameters, resulting in a substantially smaller trainable parameter budget. This produces an approximately two-order-of-magnitude reduction in the number of trainable parameters compared with full fine-tuning. Therefore, the main efficiency advantage of PEFT in this setting lies in reducing the optimization burden rather than changing the backbone architecture itself. The memory analysis shows that QLoRA_r16 achieves the lowest observed peak training GPU memory, with 1727 MB compared with 2478 MB for full fine-tuning and 2644 MB for LoRA_r16. This corresponds to an approximate reduction of 30.3% relative to full fine-tuning and 34.7% relative to LoRA_r16 under the evaluated training configuration. However, this comparison is not strictly batch-matched: QLoRA_r16 was trained with batch size 16, whereas full fine-tuning and LoRA variants were trained with batch size 32. Consequently, the observed memory reduction should be interpreted as the combined effect of 4-bit NF4 quantization and the smaller batch size, rather than as the isolated effect of quantization alone. A batch-matched memory experiment would be required to quantify the independent contribution of NF4 quantization more precisely. Inference latency shows a different trade-off. Full fine-tuning records the lowest batch-1 inference latency because it uses the original full-precision backbone without adapter or dequantization overhead. LoRA-based models introduce only moderate latency overhead, whereas QLoRA_r16 shows higher latency because 4-bit weights must be dequantized during forward propagation. Thus, QLoRA provides the strongest advantage in training-time memory reduction, while LoRA offers a more balanced compromise between parameter efficiency and inference speed. It is also important to interpret the deployment implications conservatively. The latency and memory measurements were obtained on an NVIDIA RTX PRO 6000 workstation rather than on the target embedded hardware. Therefore, these results indicate potential resource efficiency under the evaluated workstation configuration, but they do not constitute direct evidence of Jetson-class or 8 GB embedded deployment performance. Direct profiling on representative embedded GPUs, together with batch-matched memory measurements, is required before drawing hardware-specific deployment conclusions. Overall, the optimal adaptation strategy depends on the dominant practical constraint: QLoRA_r16 is preferable when training-time memory is the primary bottleneck, whereas LoRA_r16 may be more attractive when inference latency is more critical.

### 4.4. Generalization and Fold Stability

To better understand model robustness beyond average performance, we analyze generalization behavior using the train–validation macro-F1 gap together with fold-wise variability across the 5-fold cross-validation protocol. The train–validation gap values reported in Table 3 provide a simple descriptive indicator of overfitting dynamics. Full fine-tuning exhibits a slightly negative gap (−0.17), suggesting that the model fits the training distribution marginally better than the validation data. Both LoRA configurations show negative gaps (−0.41 for r = 8 and −0.27 for r = 16), indicating that low-rank adaptation changes the optimization behavior but does not eliminate training–validation differences. QLoRA_r16 behaves differently from the other methods, producing a positive gap (+0.24). This observation is compatible with a possible regularization-like empirical pattern; however, it should not be interpreted as mechanistic evidence that NF4 quantization itself improves generalization. Because the present study does not include ablation experiments that independently vary quantization, adapter rank, batch size, and optimizer dynamics, the observed pattern should be treated as configuration-level evidence only. Fold-wise standard deviations provide complementary descriptive evidence of stability, with LoRA_r16 showing the lowest variation, followed by QLoRA_r16, full fine-tuning, and LoRA_r8. These results suggest that PEFT methods can provide competitive average performance while modifying the stability profile of the fine-tuning process, but stronger causal claims require dedicated ablation studies.

## 5. Discussion

The experimental results provide a controlled within-dataset perspective on how parameter-efficient fine-tuning (PEFT), particularly LoRA and QLoRA, behaves in photovoltaic electroluminescence (EL) defect classification. When interpreted in relation to prior literature, the findings provide evidence of model capacity, class imbalance sensitivity, computational trade-offs, and validation boundaries under the specific ConvNeXt-Tiny/POLY benchmark setting. The results indicate that PEFT can match or slightly exceed full fine-tuning in macro-F1 while updating substantially fewer trainable parameters. In this study, QLoRA_r16 achieves a higher mean macro-F1 than full fine-tuning while operating with the same trainable-parameter budget as LoRA_r16. However, the absolute improvement is modest and should not be interpreted as architecture-independent superiority. Rather, the result suggests that, for this dataset and backbone, low-rank adaptation provides sufficient capacity to adapt pre-trained visual representations to PV defect classification. The observed QLoRA behavior should also be interpreted cautiously. EL-based defect classification is dominated by localized texture, intensity gradients, and structural discontinuities, and it is plausible that constrained adaptation can reduce sensitivity to training-specific patterns. Nevertheless, the present benchmark does not isolate the independent effect of NF4 quantization. Quantization, adapter rank, batch size, optimizer dynamics, and class-weighted loss all interact within the evaluated configuration. Therefore, any regularization-related explanation should be treated as a hypothesis requiring targeted ablation rather than as a conclusion established by the present experiments. The class-wise results further show that QLoRA_r16 performs well on visually ambiguous or underrepresented categories such as intact and surface-diffuse defects. This behavior is consistent with the broader observation that PEFT may alter class-wise decision behavior under imbalance. However, because the study does not separately evaluate quantization-only, LoRA-only, batch-matched QLoRA, or alternative loss-function settings, the observed class-wise changes cannot be attributed exclusively to NF4 quantization or to a specific regularization mechanism. To provide a broader perspective, Table 6 situates the proposed approach within recent PV defect classification literature. The comparison highlights a clear methodological distinction between prior work and the present study.

Several limitations should be considered when interpreting the results. First, the benchmark is restricted to a single ConvNeXt-Tiny backbone, and therefore the findings cannot be assumed to generalize directly to transformer-based, self-supervised, or lightweight architectures. Second, the experiments are conducted on the private POLY dataset, and validation on public PV defect benchmarks such as ELPV and PVEL-AD remains necessary to assess cross-dataset generalization. Third, the present study does not include ablation experiments that independently isolate the effects of NF4 quantization, adapter rank, batch size, and optimization dynamics. Fourth, the QLoRA memory comparison is not batch-matched, and the latency/memory profiling was performed on a workstation GPU rather than on embedded hardware. These limitations mean that the present findings should be interpreted as controlled within-dataset benchmark evidence rather than as general deployment-ready evidence. The results suggest that parameter-efficient fine-tuning can provide a competitive alternative to full fine-tuning for PV defect classification under the controlled POLY benchmark setting. QLoRA_r16 achieves the highest mean macro-F1 and the lowest observed peak training memory under the evaluated configuration, but the memory comparison is not batch-matched and should not be interpreted as a quantization-only advantage. Similarly, the observed class-wise behavior of QLoRA should be treated as an empirical pattern rather than as confirmed evidence of an NF4-driven regularization mechanism. Broader conclusions require additional validation on public PV benchmarks, alternative backbones, batch-matched memory profiling, direct embedded-device measurements, and dedicated ablation studies separating the effects of quantization, adapter rank, batch size, and optimization dynamics.

## 6. Conclusions

This study presents a controlled benchmark of parameter-efficient fine-tuning methods for photovoltaic defect classification. Full fine-tuning, LoRA with ranks 8 and 16, and 4-bit QLoRA with rank 16 were compared on a ConvNeXt-Tiny backbone using the 17,377-image, five-class POLY electroluminescence dataset, which preserves the natural 6.7× class imbalance. The experiments were conducted using a group-aware StratifiedGroupKFold protocol based on the available cell or panel-image identifiers, thereby reducing identifiable group leakage where metadata are available. However, because the grouping metadata are close to one-to-one and do not provide complete module-level lineage for all samples, the reported results should be interpreted as controlled within-dataset, group-aware cross-validation results rather than as fully module-independent deployment estimates. The results show that all PEFT variants slightly outperform full fine-tuning in macro-F1 while training substantially fewer parameters. QLoRA_r16 achieves the highest mean macro-F1 score of 79.92 ± 0.75%, compared with 78.26 ± 0.94% for full fine-tuning. The absolute gain is modest, but it is accompanied by improved F1 scores for the intact and surface-diffuse classes. This class-wise pattern should be interpreted as an empirical observation under the evaluated configuration rather than as confirmed evidence of an NF4-driven regularization mechanism. Dedicated ablation experiments separating quantization, adapter rank, batch size, optimizer dynamics, and loss-function effects are required before drawing a mechanistic conclusion. The efficiency analysis further shows that QLoRA_r16 obtains the lowest observed peak training GPU memory under the evaluated configuration. Because QLoRA_r16 was trained with batch size 16 whereas full fine-tuning and LoRA variants used batch size 32, this memory reduction should be interpreted as an end-to-end configuration effect rather than as the isolated effect of 4-bit NF4 quantization. Similarly, the latency and memory measurements were obtained on an NVIDIA RTX PRO 6000 workstation and therefore should not be considered direct evidence of Jetson-class or 8 GB embedded deployment performance. Direct profiling on representative embedded GPUs and batch-matched memory experiments are required before making hardware-specific deployment claims.

The findings suggest that parameter-efficient adaptation is a promising and performance-competitive alternative to full fine-tuning for PV defect classification under class imbalance and resource constraints. In the present benchmark, QLoRA_r16 is most attractive when training-time memory is the primary bottleneck, whereas LoRA_r16 provides a more balanced trade-off between parameter efficiency and inference latency. Future work should extend the benchmark to transformer-family and self-supervised backbones, including DINOv2 [38], Vision Transformer backbones [39], and ConvNeXt V2 [40], evaluate batch-matched memory and latency settings, perform direct embedded-device profiling, investigate PEFT strategies beyond the LoRA family, and assess cross-dataset generalization on public PV defect datasets such as ELPV and PVEL-AD. These extensions are necessary to determine whether the trends observed in the present controlled POLY benchmark generalize across acquisition conditions, architectures, and deployment platforms.

## Figures and Tables

**Table 1:** Distribution of the POLY dataset and class weighting strategy used in training loss.

Class	Images	Share (%)	Class Weight (1/freq)
intact	2169	12.48	1.60
cracked	1197	6.89	2.90
broken	1534	8.83	2.27
surface-diffuse	4469	25.72	0.78
surface-point	8008	46.08	0.43
Total	17,377	100.00	—

**Table 2:** Distribution of samples and groups across cross-validation folds.

Fold	Samples	Unique Groups
Fold 0	3476	3465
Fold 1	3476	3464
Fold 2	3474	3464
Fold 3	3477	3466
Fold 4	3474	3465
Total	17,377	17,324

**Table 3:** Overall comparison of full fine-tuning and PEFT methods on ConvNeXt-Tiny (mean ± std over 5 folds).

Method	Params (M)	Train %	VRAM (MB)	Latency (ms)	Accuracy (%)	F1-M (%)	Gap
full_ft	27.824	100.00	2478	2.35 ± 0.01	83.08 ± 0.80	78.26 ± 0.94	−0.17
LoRA_r8	0.530	1.87	2631	4.11 ± 0.02	83.63 ± 0.89	78.94 ± 1.03	−0.41
LoRA_r16	1.060	3.67	2644	4.11 ± 0.02	84.44 ± 0.40	79.57 ± 0.48	−0.27
QLoRA_r16	1.060	3.67	1727	7.05 ± 0.03	84.68 ± 0.63	79.92 ± 0.75	+0.24

**Table 4:** Per-class F1-score (%) across five defect categories (mean ± std).

Method	Intact	Cracked	Broken	Surface-Diffuse	Surface-Point
full_ft	62.19 ± 2.71	67.60 ± 2.34	92.48 ± 0.78	75.44 ± 1.18	93.56 ± 0.46
LoRA_r8	63.23 ± 3.08	68.27 ± 1.54	92.90 ± 1.08	76.80 ± 1.46	93.50 ± 0.47
LoRA_r16	65.19 ± 2.51	68.00 ± 1.54	92.68 ± 0.99	77.90 ± 0.95	94.11 ± 0.34
QLoRA_r16	66.94 ± 2.10	67.18 ± 1.01	93.28 ± 1.04	78.06 ± 0.54	94.13 ± 0.51

**Table 5:** Per-Class Recall Analysis.

Method	Intact	Cracked	Broken	Surface-Diffuse	Surface-Point
full_ft	63.95 ± 4.64	68.34 ± 2.66	91.86 ± 1.95	73.91 ± 1.64	93.91 ± 1.25
LoRA_r8	61.22 ± 5.89	72.26 ± 1.57	92.25 ± 2.12	77.49 ± 2.50	93.18 ± 1.56
LoRA_r16	64.46 ± 4.96	70.01 ± 2.56	92.70 ± 1.36	77.15 ± 1.43	94.50 ± 1.06
QLoRA_r16	66.48 ± 3.60	66.67 ± 2.01	93.36 ± 2.07	78.79 ± 1.89	93.92 ± 1.01

**Table 6:** Comparison with recent photovoltaic defect classification studies.

Study	Year	Backbone/ Architecture	Dataset (# Images)	Classes	Adaptation	Reported Performance
Deitsch et al. [7]	2019	VGG-19/SVM	ELPV (2624)	4	Full FT	~88% Acc
Akram et al. [8]	2019	Lightweight CNN	Private EL	2	Full FT/Scratch	98.7% Acc
Al-Waisy et al. [21]	2022	Inception-V3 + ResNet-50 fusion	ELPV (2624)	4	Full FT	95.35% Acc
Wang et al. [9]	2023	ResNet-152 + Xception + CA	Two public EL sets	multi	Full FT	—
Cao et al. [17]	2024	Improved YOLOv8-GD	EL PV modules	multi	Full FT	detection (mAP)
Tella et al. [10]	2025	8-model ensemble (incl. ViT, SENet)	ELPV (2624)	4	Full FT (ensemble)	68.36% Acc
Al-Otum [22]	2024	CNN-based (VGG/ResNet)	EL PV modules	multi	Full FT	—
Acikgoz [36]	2024	Improved YOLOv7	PV cell cracks	—	Full FT	detection (mAP)
Zhao et al. [18]	2024	PD-DETR (Transformer)	PV EL images	multi	Full FT	detection (mAP)
Zaman & Khanam [37]	2024	PV-faultNet (custom CNN)	PV cells	multi	Scratch (real-time)	91% Pr/89% Re
Mohamed et al. [19]	2025	PV-YOLOv12n + A2C2f	PVEL-AD/Roboflow	multi	Full FT	mAP@50 = 0.91
Ebied et al. [11]	2025	DenseNet/ResNet/SENet/VGG + GAN	EL PV	multi	Full FT + GAN aug.	—
This study	2026	ConvNeXt-Tiny	POLY (17,377)	5	QLoRA r = 16 (PEFT)	84.68% Acc/ 79.92% F1-M

## Data Availability

The raw POLY electroluminescence images cannot be publicly redistributed because of institutional and industrial data-sharing restrictions. To support reproducibility within these constraints, the authors can provide the train/validation/test fold indices, group identifiers used for splitting, preprocessing and augmentation configuration, training hyperparameters, per-fold result files, and figure-generation scripts upon reasonable request, subject to institutional approval.
